# Red yeast rice supplement containing silica nanoparticles induces renal injury in rats with unilateral nephrectomy

**DOI:** 10.1007/s10157-025-02770-0

**Published:** 2025-09-22

**Authors:** Makoto Abe, Nobuyuki Magome, Yasuhiro Horibata, Tadayuki Ogawa, Akihiro Tojo

**Affiliations:** 1 Department of Nephrology and Hypertension, Blood Purification Center, Dokkyo Medical University, 880 Kitakobayashi, Mibu, Tochigi, 321-0293 Japan; 2 Department of Fundamental Education, Dokkyo Medical University, Tochigi, Japan; 3 Department of Biochemistry, Dokkyo Medical University, Tochigi, Japan; 4 Laboratory for Molecular Pathobiology, Research Center for Advanced Medical Science, Dokkyo Medical University, Tochigi, Japan

**Keywords:** Silica nanoparticle, Puberulic acid, Proximal tubule, Acute kidney injury

## Abstract

**Background:**

Acute kidney injury (AKI) caused by red yeast rice Cholestehelp® (CP) tablets has become a public health issue in Japan. Puberulic acid (PA) contaminated in CP tablets may cause AKI; however, we detected silica nanoparticles in a CP patient. CP-related kidney injury was examined in rats that underwent left nephrectomy to increase silica nanoparticle loading.

**Methods:**

Six male Sprague–Dawley rats were administered CP and underwent left nephrectomy on day 4. Blood and urine samples were collected on day 11. Renal tissues were observed by electron microscopy and low-vacuum scanning electron microscopy-energy dispersive X-ray spectroscopy (LVSEM-EDS). The amount of PA in CP was measured, and PA was administered to normal rats and unilaterally nephrectomized rats.

**Results:**

Normal rats receiving CP (2KCP) had increased urine volume and lower urine specific gravity than controls, but no significant changes were observed in urinary protein, renal function, electrolytes, or blood gasses. Unilaterally nephrectomized rats receiving CP (1KCP) had increased water intake and urine volume, decreased urine specific gravity, and increased low-molecular-weight proteinuria. The glomeruli of 1KCP rats showed expanded subendothelial space and increased endocytic vesicles were observed in the proximal tubules relative to 2KCP rats. The accumulation of nanoparticles in the endosomes of the proximal tubules, and LVSEM-EDS detected silicon in renal tissue. Administration of PA at the doses in CP tablet did not result in significant renal injury.

**Conclusions:**

Uninephrectomized rats administered CP tablets showed accumulation of silicon-containing nanoparticles in the proximal tubules and renal injury.

## Introduction

Red yeast rice (RYR) is a dietary supplement whose component monacolin has been shown to have cholesterol-lowering effects, and its safety has been proven [1–3]. In Japan, kidney dysfunction caused by Kobayashi Pharmaceutical’s RYR Cholestehelp® (CP) has become a social issue, and according to a survey by the Japanese Society of Nephrology, approximately 192 patients developed acute kidney injury (AKI) accompanied by Fanconi syndrome, with a few cases requiring maintenance dialysis [4] Some of them have been individually reported [5–21]. Puberulic acid (PA) and two other compounds produced by blue mold contaminating RYR have been identified in CP tablets [21]; however, the PA concentrations in CP tablets have not been reported to date. There have been reports from China investigating detection methods for PA by mixing it with CP tablets [22]. We reported a case of CP-induced AKI in which silicon-containing nanoparticles were detected in the proximal tubules [13]. CP tablets are taken by many people with high cholesterol levels, but only approximately two hundred patients have developed AKI [4]. We hypothesized that CP tablets containing silica nanoparticles would be filtered in the glomerulus and endocytosed in the proximal tubules when administered to patients with proteinuria, leading to AKI. Therefore, we administered CP tablets to normal rats (2KCP) and unilaterally nephrectomized rats (1KCP).

## Material and methods

### Experimental animals

Eighteen 7-week-old male Sprague–Dawley rats with 200 g body weight (CLEA Japan, Tokyo, Japan) were used in the experiment and individually housed in rat cages. One tablet (200 mg) of Cholestehelp® (CP, Lot # X3017, exp.2025.10.15) crushed with a mortar and pestle and dissolved in 2.5 mL of saline (1 Tab 200 mg/rat body weight 200 g/5 days, 200 mg/kg/day), which is 20 times the human dosage (3T 600 mg/human body weight 60kg/day, 10 mg/kg/day). Up to day 11, 0.5 mL of CP solution was administered orally once daily using a metal oral probe. The left kidney was nephrectomized on day 4, the right kidney was removed on day 11, and the rats were sacrificed. Drinking water and urine volumes were measured using metabolic cages before CP administration, the day before left nephrectomy, and the day before right nephrectomy. The control rats were administered saline (0.5 mL) without CP. This study received ethical approval from the Dokkyo Medical University Animal Experiment Committee and was conducted in compliance with the Dokkyo Medical University Animal Experiment Regulations (approval number: 1506).

During surgery, a triple combination of medetomidine hydrochloride 0.4 mg/kg (Nihon Zenyaku Kogyo, Fukushima, Japan), midazolam 2.0 mg/kg (Maruishi Pharmaceutical, Osaka, Japan), and propofol 2.5 mg/kg (Meiji Animal Health, Kumamoto, Japan) was administered intraperitoneally, followed by the administration of isoflurane inhalation anesthetic solution 2.5–5.0% (Viatris Pharmaceutical, Tokyo, Japan). The renal artery, renal vein, and ureter of the left kidney were ligated, and the left kidney was removed and used for morphological examination. After the left nephrectomy, the peritoneum, muscle, and skin were sutured, and the rats were returned to their cages. On day 11, blood samples were collected from the inferior vena cava, and the right kidney was removed before sacrifice.

To confirm the effect of PA contaminated on the CP table, we conducted additional experiments with four groups: rats with normal two kidneys that received PA drink (2KPA) or tap water (2KC), and rats that underwent left nephrectomy that received PA drink (1KPA) or tap water (1KC). As shown later, the CP tablet contained 0.84 μg of PA; each rat was given 0.168 μg/day of PA (Nagara Science Co. Ltd., Gifu, Japan) in drinking water (50 mL/day) for 5 days. The urine samples were collected using metabolic cages, and then the rats were anesthetized, and blood and kidney samples were obtained as described above.

### Measurement of urinary protein fractions and blood samples

Urine collected in metabolic cages was centrifuged, and the supernatant was used to measure the specific gravity using a refractometer. As described previously [10], urine samples were mixed with SDS buffer, loaded onto a 4–10% gel (Daiichi Chemicals, Tokyo, Japan), and separated by sodium dodecyl sulfate (SDS)-polyacrylamide gel electrophoresis (PAGE). After SDS-PAGE, the gels were stained with Coomassie blue. Densitometry was performed using ImageJ (NIH, Bethesda, USA), and the relative amounts of protein fractions were measured from the density and area of each band of molecular weight.

Serum albumin, creatinine, urea nitrogen, electrolytes, and blood gas were measured using a Dri-Chem NX600 (Fujifilm, Tokyo, Japan) and an i-STAT system 1 analyzer (Abbott Japan, Tokyo, Japan).

### Optical and electron microscopy

The excised kidneys were fixed in formalin, embedded in paraffin, stained with Azan or periodic acid-Schiff (PAS), and observed under a dark-field microscope. A portion of the kidney was fixed in 2.5% glutaraldehyde, post-fixed in 1% osmium tetroxide, and embedded in an epoxy resin. Ultrathin sections were stained with uranyl acetate and lead citrate and observed under a transmission electron microscope HT7800 (Hitachi High-Tech, Tokyo, Japan).

### Elemental analysis

A portion of the kidney was snap-frozen, 4-μm sections were placed on aluminum conductive tape, and an elemental analysis was performed using a low-vacuum scanning electron microscope (LVSEM) TM3000 (Hitachi, Tokyo, Japan) equipped with an energy-dispersive X-ray spectrometer (EDS) Quantax70EDS (Bruker, Madison, WI, USA), as previously described [13]. Images were taken at 2000 × magnification and an accelerating voltage of 15.0 kV. The elemental composition was analyzed from the EDS spectra using the Quantax70 software program.

### Quantification of PA by liquid-chromatography-tandem mass spectrometry (LC–MS/MS)

PA was extracted from one tablet of supplement (200 mg) dissolved in 0.3 mL of methanol as described previously [13] and quantified by reverse-phase HPLC using an L-column 3 ODS column (3 µm, 2.0 × 50 mm) (Chemicals Evaluation and Research Institute, Tokyo, Japan) coupled to a 5500 QTRAP mass spectrometer (Sciex Inc., Framingham, MA). A binary gradient consisting of solvent A (water containing 5 mM ammonium acetate and 0.04% ammonium hydroxide) and solvent B (acetonitrile containing 5 mM ammonium acetate and 0.04% ammonium hydroxide) was used. The gradient profile was: 0–2 min, 100% A; 2–5 min, 0–100% B linear gradient; 5–7 min, 95% B. The flow rate was 0.3 ml/min and the column temperature was 35 °C. PA was detected in negative ion mode with m/z transitions at 197/125 and quantified using MultiQuant (ver. 2.0, Sciex, Inc., Framingham, MA).

## Results

**Fig. 1:**
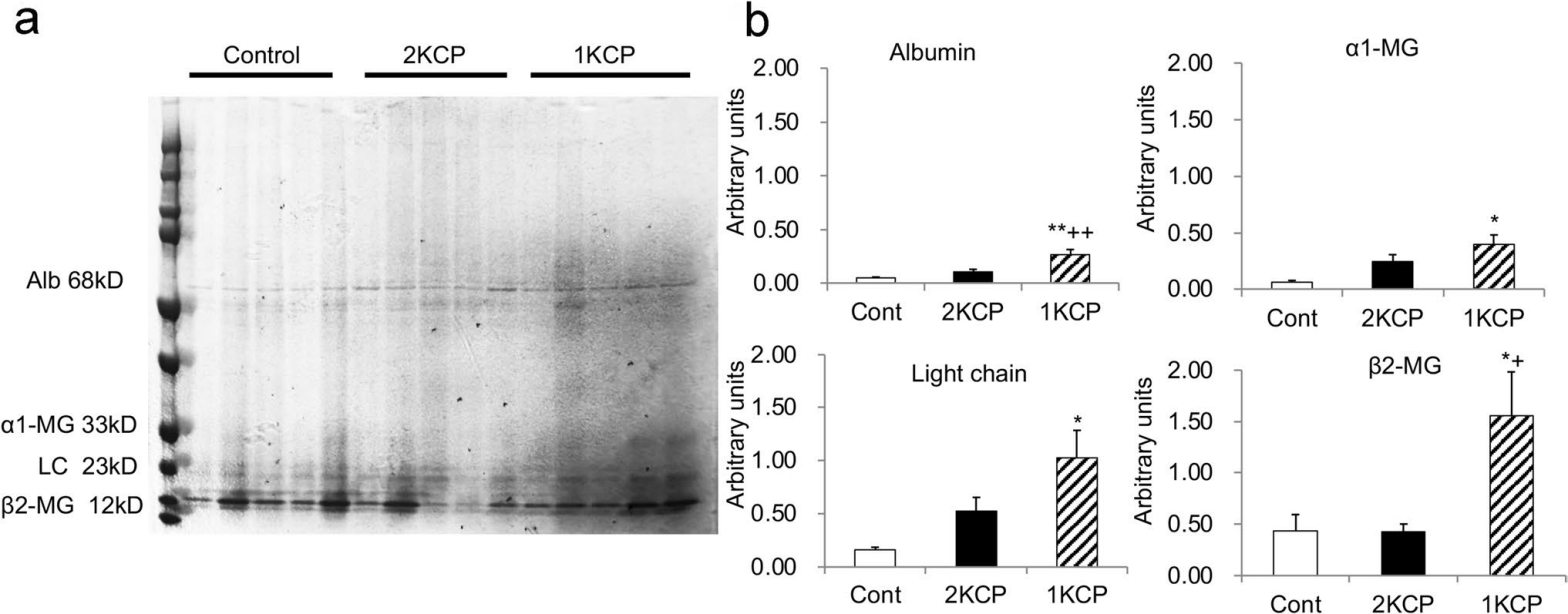
A urinary protein analysis with SDS–polyacrylamide electrophoresis and densitometry to determine the amounts of albumin, α1-microglobulin (α1-MG), light chain (LC) and β2-microglobulin (β2-MG). *p < 0.05, **p < 0.01 vs. Control, ^+^*p* < 0.05. ^++^*p* < 0.01 vs. 2KCP

In CP-treated rats with normal kidneys (2KCP), urine volume increased in comparison to the control group, but no significant changes were observed in renal function, electrolytes, or blood gasses. In CP-treated unilaterally nephrectomized rats (1KCP), water intake and urine volume increased, urine specific gravity decreased, and renal function significantly decreased relative to the control group or 2KCP (Table 1). The analysis of urinary protein fractions showed that albumin, α1-microglobulin (α1-MG), light chain (LC), and β2-microglobulin (β2-MG) did not differ to a statistically significant extent between the control group and the 2KCP group, while the values were significantly increased in the 1KCP group (Fig. 1).

**Table 1:** Physiological data of control rats, rats receiving Cholesterol Help (CP) tablets (2KCP) and unilaterally nephrectomized rats receiving CP (1KCP) at metabolic cage

	Control (*n* = 6)	2KCP (*n* = 6)	1KCP (*n* = 6)
Water intake (mL/day)	37 ± 3	47 ± 2	67 ± 5**^++^
Urine (mL/day)	10 ± 1	16 ± 2*	26 ± 1**^++^
Specific gravity	1.076 ± 0.006	1.064 ± 0.004*	1.049 ± 0.002**
Serum albumin (g/dL)	3.0 ± 0.2	3.1 ± 0.04	3.4 ± 0.1
Serum Creatinine (mg/dL)	0.24 ± 0.02	0.22 ± 0.01	0.43 ± 0.04*^++^
Blood urea nitrogen (mg/dL)	21.1 ± 2.2	16.6 ± 0.6	26.7 ± 1.8^++^
Serum Na (mEq/L)	138.3 ± 0.7	138.5 ± 0.76	138.1 ± 0.6
Serum K (mEq/L)	4.6 ± 0.2	4.5 ± 0.05	5.3 ± 0.5
Serum Ca (mg/dL)	10.0 ± 0.1	10.3 ± 0.2	10.9 ± 0.3
Serum P (mg/dL)	10.5 ± 0.5	10.9 ± 0.4	10.4 ± 0.4
Blood pH	7.413 ± 0.022	7.441 ± 0.013	7.381 ± 0.016
PCO_2_ (mmHg)	43.5 ± 2.7	41.2 ± 1.3	52.0 ± 2.6
HCO_3_^−^ (mEq/L)	27.7 ± 0.5	28.0 ± 0.3	30.6 ± 0.8*

*p < 0.005, **p < 0.001 vs. Control, ^+^*p* < 0.05, ^++^*p* < 0.01 vs. 2KCP

**Fig. 2:**
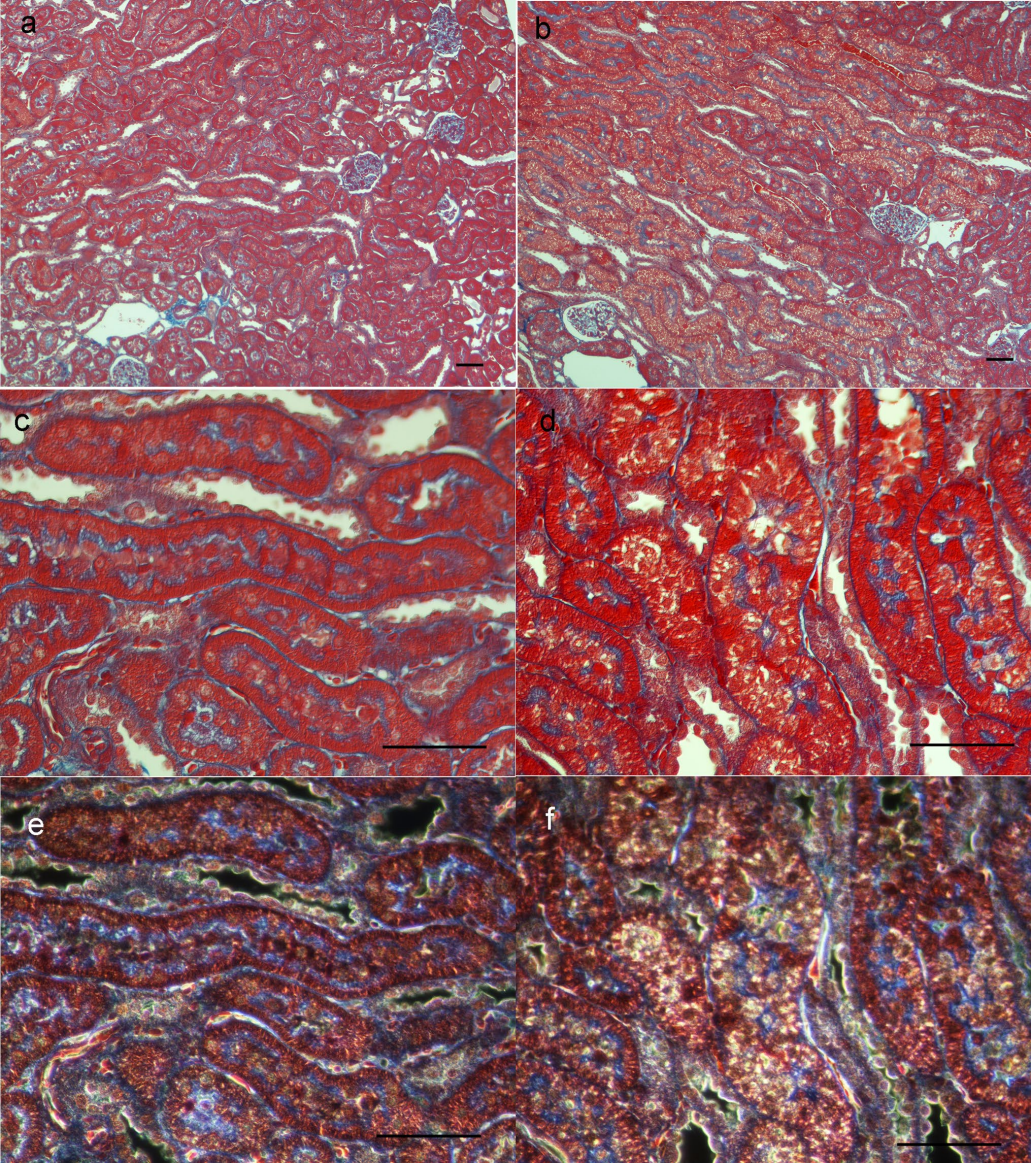
AZAN staining of the kidneys of normal rats treated with CP tablets (2KCP; **a**, **c**, **e**) and left nephrectomized rats treated with CP tablets (1KCP; **b**, **d**, **f**). Proximal tubules **c**, **d**) were observed in the same area on dark field images (**e**, **f**). The bar indicates 50 μm

**Fig. 3:**
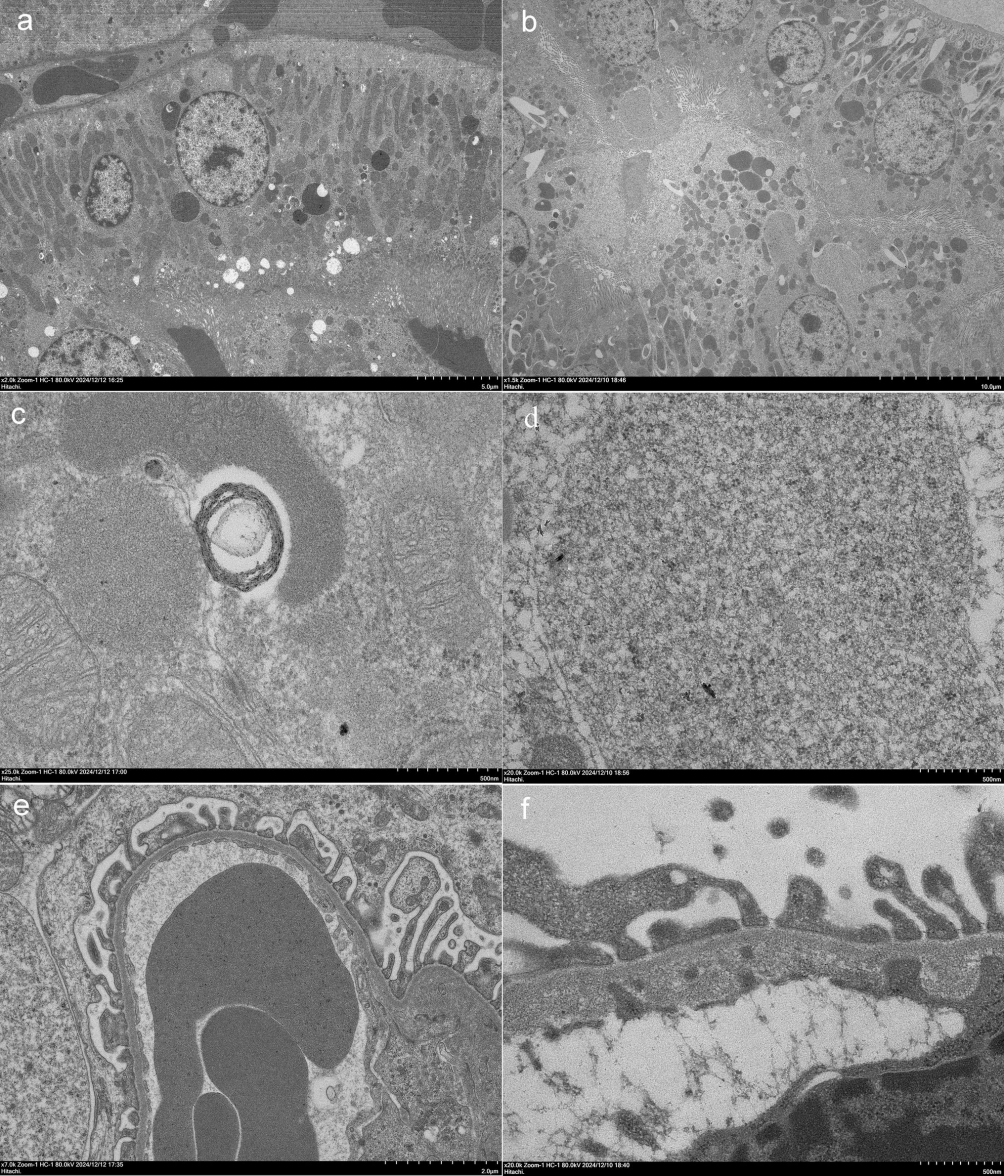
Electron microscopy of the kidneys of normal rats treated with CP tablets (2KCP; **a**, **c**, **e**) and left nephrectomized rats treated with CP tablets (1KCP; **b**, **d**, **f**)

As no significant differences were found in blood tests or urinary protein levels between the control and 2KCP groups, the following histological examinations compared 2KCP and 1KCP. AZAN staining showed that the endocytic vesicles in the proximal tubules were more prominent in the 1KCP group than in the 2KCP group, and dark-field microscopy revealed a large accumulation of birefringent material within the endocytic vesicles (Fig. 2). TEM observations revealed 10-nm black granules in the tubular vesicles of 1KCP (Fig. 3b, d), but only a few were observed in 2KCP (Fig. 3a, c). The glomeruli were normal in 2KCP (Fig. 3e), but the subendothelial space was enlarged and nanoparticles were observed in 1KCP (Fig. 3f).

**Fig. 4:**
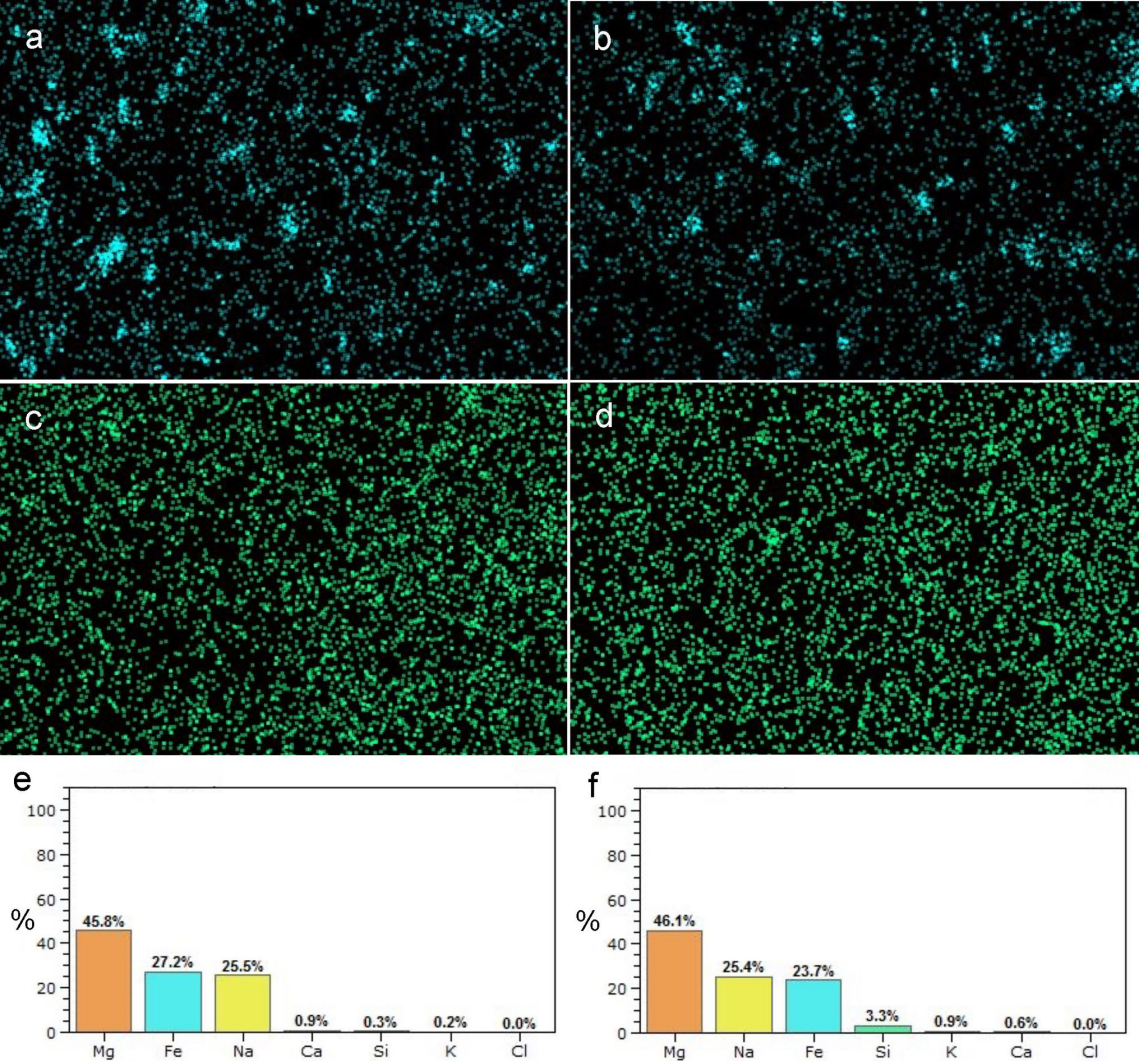
The element analysis of the kidneys of normal rats treated with CP tablets (2KCP; **a**, **c**, **e**) and left nephrectomized rats treated with CP tablets (1KCP; **b**, **d**, **f**) illustrating Fe (**a**, **b**), Si (**c**, **d**) and molecular weight % (**e**, **f**)

The elemental analysis using LVSEM-EDS revealed that silicon (Si) was the fourth most abundant element in the renal cortex, with 1KCP rats containing 3.0% Si, which was 10 times higher than the concentration found in 2KCP rats (0.3%) (Fig. 4).

**Fig. 5:**
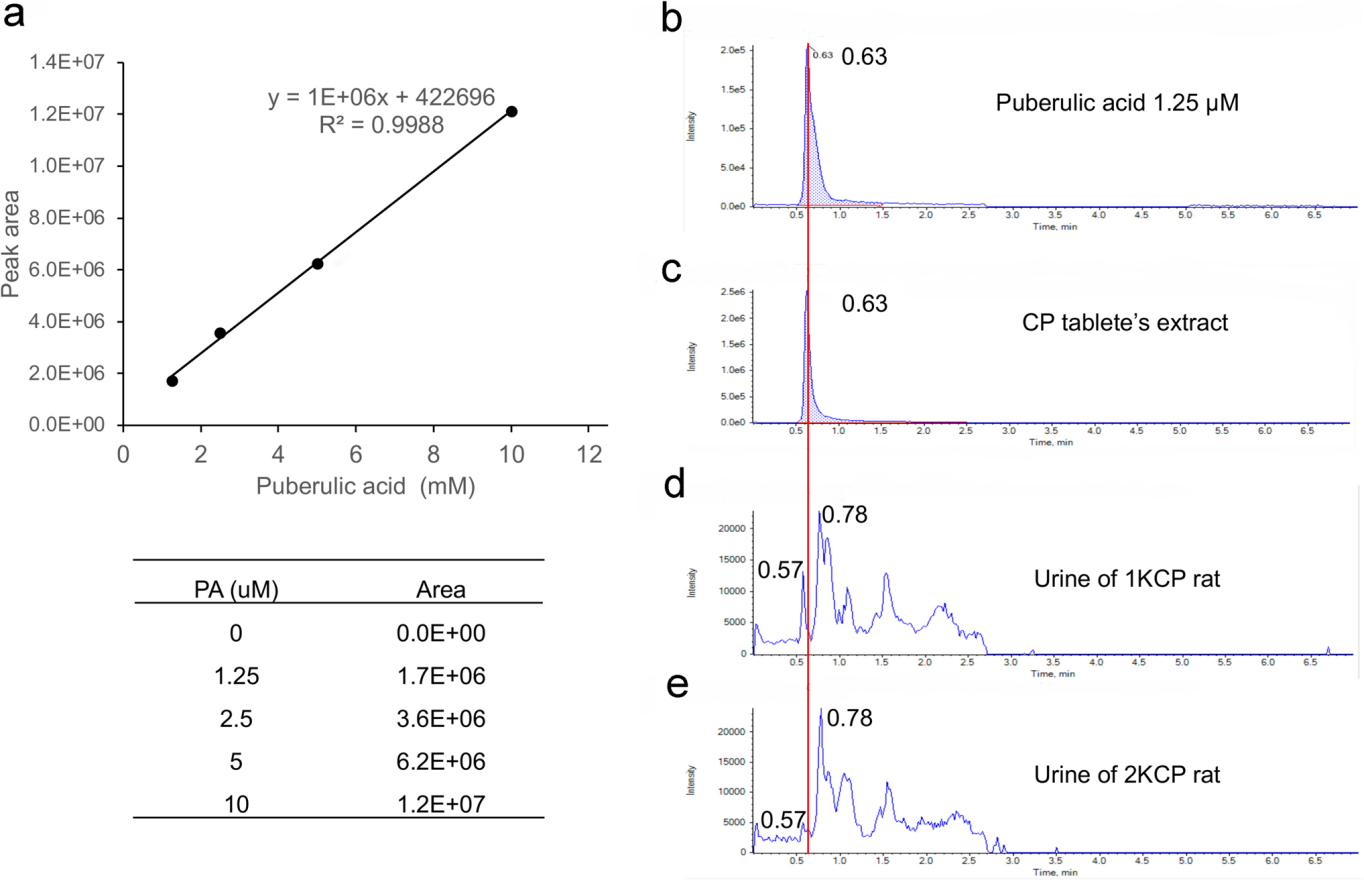
Standard curve and representative chromatograms of LC–MS/MS. Standard curve plotting the peak area against the PA concentration (**a**). Chromatograms of 1.25 μM of standard PA (**b**), CP tablet extract (**c**), urine of 1KCP rats (**d**), and urine of 2KCP rats (**e**). The peak corresponding to PA was detected at a retention time of 0.63 min. No corresponding peaks were observed for the urine samples

The LC–MS/MS analysis of the CP tablet extract revealed that PA concentration was 14 μM, indicating one CP tablet contained 0.84 μg of PA. PA has a small molecular weight of 198 Da and should be filtered in the glomerulus; however, PA was not detected in the urine of rats (Fig. 5).

**Fig. 6:**
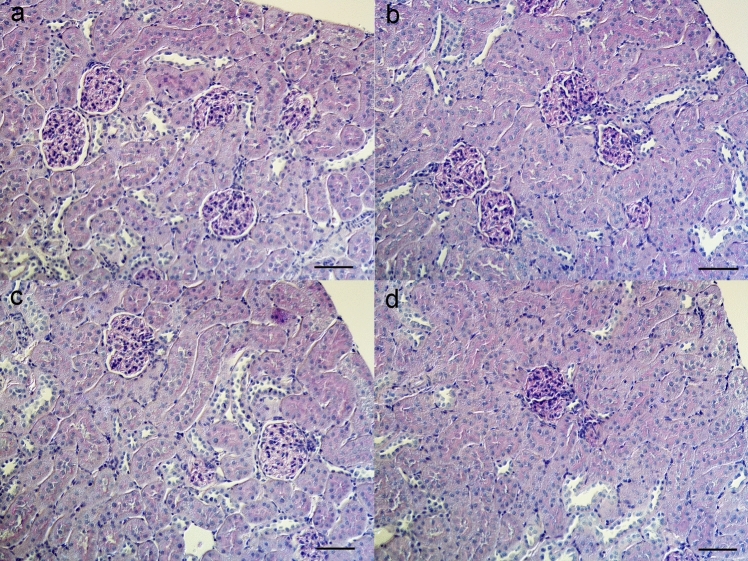
PAS staining of the kidneys of normal control rats received tap water (2KC, **a**) or puberulic acid drink (2KPA, **b**), rats underwent left nephrectomy received tap water (1KC, **c**) or PA drink (1KPA, **d**). The bar indicates 50 μm

Physiological data of normal control rats received tap water (2KC) or puberulic acid drink (2KPA); rats that underwent left nephrectomy received tap water (1KC) or PA drink (1KPA) are shown in Table 2. Compared with 2KC, 1KC significantly increased serum creatinine and decreased urine specific gravity, but no significant changes were observed between 2KPA and 2KC or 1KPA and 1KC. Urinary excretion of albumin and β2-microglobulin did not show a significant change by PA administration (Table 2). Morphologically, PAS staining did not show significant changes in glomeruli and renal tubules by PA administration (Fig. 6).

**Table 2:** Physiological data of normal control rats received tap water (2KC) or puberulic acid drink (2KPA), rats underwent left nephrectomy received tap water (1KC) or PA drink (1KPA) at metabolic cage

	2KC (*n* = 3)	2KPA (*n* = 3)	1KC (*n* = 3)	1KPA (*n* = 3)
Water intake (mL/day)	46 ± 1	74 ± 13	59 ± 4	35 ± 5 +
Urine (mL/day)	21 ± 3	36 ± 9	31 ± 4	14 ± 2
Specific gravity	1.069 ± 0.013	1.032 ± 0.010	1.030 ± 0.004*	1.056 ± 0.004
Serum albumin (g/dL)	3.1 ± 0.1	3.2 ± 0.1	2.6 ± 0.3	2.5 ± 0.4
Serum Creatinine (mg/dL)	0.24 ± 0.02	0.28 ± 0.01	0.47 ± 0.08* +	0.40 ± 0.02
Blood urea nitrogen (mg/dL)	20.9 ± 2.3	19.2 ± 3.1	29.4 ± 7.3	20.9 ± 1.4
Serum Na (mEq/L)	143.0 ± 2.0	138.7 ± 0.9	137.0 ± 2.3	135.0 ± 2.3
Serum K (mEq/L)	4.8 ± 0.2	4.8 ± 0.3	5.1 ± 0.4	4.6 ± 0.1
Serum Ca (mg/dL)	9.8 ± 0.1	9.8 ± 0.2	9.0 ± 0.4	8.8 ± 0.4
Serum P (mg/dL)	10.1 ± 0.4	10.4 ± 0.3	8.0 ± 0.2 +	8.4 ± 0.8
Blood pH	7.400 ± 0.013	7.419 ± 0.002	7.446 ± 0.011	7.441 ± 0.002
PCO_2_ (mmHg)	44.6 ± 2.2	45.2 ± 0.1	45.0 ± 1.0	46.7 ± 6.8
HCO_3_^−^ (mEq/L)	27.6 ± 0.5	29.2 ± 0.1	30.7 ± 0.4	27.4 ± 2.3
Urinary albumin (A.U)	0.38 ± 0.04	0.34 ± 0.02	0.51 ± 0.15	0.40 ± 0.08
Urinary β2-MG (A.U)	0.75 ± 0.35	0.95 ± 0.27	0.69 ± 0.05	0.63 ± 0.13

*AU* arbitrary unit

*p < 0.005 vs. 2KC, ^+^*p* < 0.05 vs. 2KPA

## Discussion

In this study, renal injury due to CP tablets was observed in unilaterally nephrectomized rats, and silicon-containing nanoparticles were identified in the proximal tubules, similar to a case report in humans [13]. The CP tablets used were the same as those used by the patient, and PA was detected in the tablets, as reported by Kobayashi Pharmaceutical Co. Ltd. However, in normal rats administered CP tablets (2KCP), there was little renal injury, abnormal urinary protein, renal tissue injury, or silicon-including nanoparticle uptake. A national survey found that 10% of AKI patients had kidney disease, 20% had hypertension, 5% had diabetes, and 10% of patients showed glomerular lesions on a renal biopsy [4], including membranous nephropathy [6], suggesting that individuals who already have glomerular lesions that filter nanoparticles may be more susceptible to developing AKI, as in animal studies of 1KCP rats. Silica nanoparticles are used as safe additives for drug stability and delivery; however, silica nanoparticles can damage organs, including the kidneys and liver [23–25]. The results of this animal study confirm the mechanism of proximal tubular damage via silicon-including nanoparticles observed in our clinical case [13] and the reported case with electron-dense particles in the proximal tubule [7]. In our clinical case report [13], we have demonstrated that increased mitochondria ROS production is one of the mechanisms of proximal tubular damage. In 1KCP rats, EM observations showed that excess filtered nanoparticles were not metabolized but accumulated in the endosomes and lysosomes of the proximal tubules (Fig. 3), and these particles could directly cause morphological damage to the proximal tubules.

PA is known as an anti-malarial drug, and PA derivatives at an oral dose of 15 mg/kg showed potent antiparasitic effects without any apparent toxicity [26]. PA also exhibited weak cytotoxicity against human MRC-5 cells, with an IC_50_ value of 57.2 µg/ml [27], which was higher than the PA concentration contaminated in CP tablets (Fig. 5). Although the albumin-binding capacity of PA is not known, some free PA may exist and pass through the glomerulus, causing tubular damage in both 2KCP and 1KCP rats. However, no significant tubular damage was observed in the 2KCP group, suggesting that PA was not the primary cause of CP-induced AKI. Even though PA was detected in CP tablets at relatively high concentrations (Fig. 5), it could not be detected in the urine of either 2KCP or 1KCP rats. The amount of PA contained in a CP tablet was only 0.84 μg; thus, urinary PA concentrations may be below the sensitivity of LC–MS/MS detection. At the same doses of PA contained in CP tablets, no significant changes in renal function or proteinuria were observed between 2KC and 2KAP or between 1KC and 1KPA (Table 2, Fig. 6). Therefore, PA may not be the main cause of renal injury due to CP tablet, but further investigation is needed. The precise mechanism of cytotoxicity of CP has not yet been clarified, but recently it was reported that the CP tablets induced apoptosis of adult kidney stem cell organoids in vitro [19]. We first demonstrated proximal tubular damage by silica nanoparticles in CP tablets in an in vivo animal study.

### Limitations

This study attempted to investigate kidney damage caused by the PA-contaminated CP tablets and did not investigate the general adverse effects of silica contained in various functional foods containing CP. (i) No group is exposed to silica only, (ii) LVSEM-EDS detects elemental Si and therefore does not specifically identify SiO₂. (iii) Because PA was administered at one concentration for only a short period of time, PA-induced kidney injury cannot be completely excluded.

## Conclusion

In unilaterally nephrectomized rats administered CP tablets, silicon-containing nanoparticles cross the glomerular filtration barrier and accumulate in proximal tubule cells, but not in normal rats administered CP tablets.

## References

[1] Heber D, Yip I, Ashley JM, Elashoff DA, Elashoff RM, Go VLW. Cholesterol-lowering effects of a proprietary Chinese red-yeast-rice dietary supplement. Am J Clin Nutr. 1999;69(2):231–6. 9989685 10.1093/ajcn/69.2.231

[2] Trogkanis E, Karalexi MA, Sergentanis TN, Kornarou E, Vassilakou T. Safety and efficacy of the consumption of the nutraceutical “red yeast rice extract” for the reduction of hypercholesterolemia in humans: a systematic review and meta-analysis. Nutrients. 2024. 10.3390/nu16101453. 38794691 10.3390/nu16101453 PMC11124448

[3] Cho KH, Bahuguna A, Kim JE, Lee SH, Lee Y, Jeon C. A comparative effect of 12-week dietary intervention of policosanol (Raydel®) and red yeast rice (RYR, Kobayashi) in managing dyslipidemia and organ damage in hyperlipidemic zebrafish. Pharmaceuticals. 2025;18(2):200. 10.3390/ph18020200. 40006014 10.3390/ph18020200 PMC11859080

[4] Shinzawa M, Matsui I, Doi Y, Matsumoto A, Takahashi A, Nangaku M, et al. A nationwide questionnaire study evaluated kidney injury associated with Beni-koji tablets in Japan. Kidney Int. 2025;107(3):530–40. 10.1016/j.kint.2024.11.027. 39708997 10.1016/j.kint.2024.11.027

[5] Habuka M, Hosojima M, Yata Y, Kurumada K, Yamagiwa M, Yonezawa M, et al. Fanconi syndrome with acute proximal tubular injury induced by a dietary supplement containing Beni-Koji: a case series report. BMC Nephrol. 2024;25(1):466. 10.1186/s12882-024-03903-5. 39639196 10.1186/s12882-024-03903-5 PMC11622456

[6] Iwamura N, Tsutsumi K, Yamada S, Uesugi N, Hamashoji T, Arita Y, et al. Subclinical acute tubular necrosis potentially associated with red yeast rice consumption unexpectedly detected in a patient with membranous nephropathy. CEN Case Rep. 2024. 10.1007/s13730-024-00946-3. 39560710 10.1007/s13730-024-00946-3 PMC12307268

[7] Kawai Y, Ozawa M, Isomura A, Mitsuhashi H, Yamaguchi S, Nagayama S, et al. A case of Fanconi syndrome that developed following a year of consumption of a red yeast rice supplement. CEN Case Rep. 2024. 10.1007/s13730-024-00913-y. 38985380 10.1007/s13730-024-00913-y PMC11785878

[8] Miyazaki R, Takahashi Y, Katayama Y, Kawamura T, Tsuboi N, Yokoo T. Tubular glycogen accumulation in acute kidney injury associated with red yeast rice supplement. Clin Kidney J. 2024;17(11):sfae318. 10.1093/ckj/sfae318. 39559171 10.1093/ckj/sfae318 PMC11570830

[9] Miyazaki R, Takahashi Y, Kawamura T, Ueda H, Tsuboi N, Yokoo T. Acute kidney tubular injury after ingestion of red yeast rice supplement. Clin Kidney J. 2024;17(6):sfae151. 10.1093/ckj/sfae151. 38846105 10.1093/ckj/sfae151 PMC11153872

[10] Murata Y, Hemmi S, Akiya Y, Miyasato K, Kobayashi H, Maruyama T, et al. Certain red yeast rice supplements in Japan cause acute tubulointerstitial injury. Kidney Int Rep. 2024;9(9):2824–8. 10.1016/j.ekir.2024.06.022. 39291198 10.1016/j.ekir.2024.06.022 PMC11403028

[11] Oda K, Murata T, Tanaka F, Oda H, Tsujimoto K, Fukumori A, et al. A case of acute kidney injury and Fanconi syndrome while taking multiple supplements, including Red Yeast Rice Cholesterol Help(®). CEN Case Rep. 2024. 10.1007/s13730-024-00903-0. 38900361 10.1007/s13730-024-00903-0 PMC11785839

[12] Uchiyama K, Otani M, Chigusa N, Sugita K, Matsuoka R, Hosoya K, et al. Acute kidney injury associated with red yeast rice (Beni-Kōji) supplement: a report of two cases. Kidney Med. 2024;6(11):100908. 10.1016/j.xkme.2024.100908. 39507393 10.1016/j.xkme.2024.100908 PMC11539353

[13] Abe M, Ogawa T, Magome N, Ono Y, Tojo A. Element analysis applied to investigate acute kidney injury induced by red yeast rice supplement. Med Mol Morphol. 2025;58(1):53–61. 10.1007/s00795-024-00411-1. 39535557 10.1007/s00795-024-00411-1 PMC11829840

[14] Doi T, Shimizu A, Morimoto E, Morii K, Okubo A, Mizuiri S, et al. A case of acute kidney injury and fanconi syndrome caused by a red yeast rice supplement. Internal Med. 2025. 10.2169/internalmedicine.4330-24. 10.2169/internalmedicine.4330-24 PMC12425577 39993755

[15] Maruyama H, Sada KE, Oka M, Yanai M, Hidaka S, Kobayashi S. Successful resolution of suspected red yeast rice-induced nephropathy. Intern Med. 2025. 10.2169/internalmedicine.4908-24. 40090717 10.2169/internalmedicine.4908-24 PMC12463421

[16] Shimokawa M, Kajio Y, Kawanishi K, Kawanishi K, Shiomi M, Morikawa T, et al. Acute tubular injury and Fanconi syndrome associated with red yeast rice supplement. Kidney Int Rep. 2025;10(3):956–9. 10.1016/j.ekir.2024.12.016. 40225362 10.1016/j.ekir.2024.12.016 PMC11993213

[17] Ushimaru S, Tominaga N. Acute kidney injury with Fanconi syndrome following intake of a red yeast rice supplement. Kidney Int Rep. 2025;10(1):269–70. 10.1016/j.ekir.2024.11.010. 39810787 10.1016/j.ekir.2024.11.010 PMC11725804

[18] Chikasue A, Taguchi K, Iwatani R, Kimura K, Okuda S, Uesugi N, et al. Three cases of red yeast rice-containing supplement-induced acute kidney injury and Fanconi syndrome. Am J Kidney Dis. 2025;85(4):522–6. 10.1053/j.ajkd.2024.08.007. 39424254 10.1053/j.ajkd.2024.08.007

[19] Nakanoh H, Tsuji K, Fukushima K, Haraguchi S, Kitamura S, Wada J. Supplement-induced acute kidney injury reproduced in kidney organoids. Am J Nephrol. 2025. 10.1159/000544795. 39978331 10.1159/000544795 PMC12342697

[20] Kamada A, Hirose T, Hashimoto H, Konya Y, Sato F, Ishiyama K, et al. Tubular damage and SGLT2 expression in a patient with Beni-koji tablet-associated acute kidney injury and Fanconi syndrome. CEN Case Rep. 2025. 10.1007/s13730-025-00984-5. 40126859 10.1007/s13730-025-00984-5 PMC12126451

[21] Tanaka S, Masumoto N, Makino T, Matsushima Y, Morikawa T, Ito M. Novel compounds isolated from health food products containing beni-koji (red yeast rice) with adverse event reports. J Nat Med. 2024;78(4):845–8. 10.1007/s11418-024-01827-w. 38834898 10.1007/s11418-024-01827-w

[22] Pan HQ, Feng R, Tan YN, Qin XY, Cao YM, Mao XH, et al. Determination of puberulic acid in monascus-fermented red yeast rice by LC-MS/MS combined with precolumn derivatization. Toxins. 2025;17(1):11. 10.3390/toxins17010011. 10.3390/toxins17010011 PMC11769100 39852964

[23] Bolton WK, Suratt PM, Strugill BC. Rapidly progressive silicon nephropathy. Am J Med. 1981;71(5):823–8. 10.1016/0002-9343(81)90374-0. 6272574 10.1016/0002-9343(81)90374-0

[24] Mahmoud AM, Desouky EM, Hozayen WG, Bin-Jumah M, El-Nahass ES, Soliman HA, et al. Mesoporous silica nanoparticles trigger liver and kidney injury and fibrosis via altering TLR4/NF-κB, JAK2/STAT3 and Nrf2/HO-1 signaling in rats. Biomolecules. 2019. 10.3390/biom9100528. 31557909 10.3390/biom9100528 PMC6843412

[25] Niroumand U, Firouzabadi N, Goshtasbi G, Hassani B, Ghasemiyeh P, Mohammadi-Samani S. The effect of size, morphology and surface properties of mesoporous silica nanoparticles on pharmacokinetic aspects and potential toxicity concerns. Front Mater. 2023;10:1189463. 10.3389/fmats.2023.1189463.

[26] Saito R, Sennari G, Nakajima A, Kimishima A, Iwatsuki M, Ishiyama A, et al. Discoveries and syntheses of highly potent antimalarial troponoids. Chem Pharm Bull. 2021;69(6):564–72. 10.1248/cpb.c21-00132. 10.1248/cpb.c21-00132 34078803

[27] Iwatsuki M, Takada S, Mori M, Ishiyama A, Namatame M, Nishihara-Tsukashima A, et al. *In vitro* and *in vivo* antimalarial activity of puberulic acid and its new analogs, viticolins A–C, produced by sp FKI-4410. J Antibiot. 2011;64(2):183–8. 10.1038/ja.2010.124. 10.1038/ja.2010.124 21063422

